# Time-Dependent Changes in Risk of Progression During Use of Bevacizumab for Ovarian Cancer

**DOI:** 10.1001/jamanetworkopen.2023.26834

**Published:** 2023-08-02

**Authors:** Shiro Takamatsu, Hidekatsu Nakai, Ken Yamaguchi, Junzo Hamanishi, Masaki Mandai, Noriomi Matsumura

**Affiliations:** 1 Department of Gynecology and Obstetrics, Kyoto University Graduate School of Medicine, Kyoto, Japan; 2 Department of Obstetrics and Gynecology, Kindai University Faculty of Medicine, Osaka, Japan

## Abstract

**Importance:**

Although bevacizumab has been used in the treatment of ovarian cancer, its optimal use is unknown.

**Objective:**

To investigate time-dependent changes in the outcomes of bevacizumab therapy.

**Design, Setting, and Participants:**

This cohort study was conducted using published data from 7 previous randomized phase 3 clinical trials with bevacizumab (ICON7, GOG-0218, BOOST, GOG-0213, OCEANS, AURERIA, and MITO16B) from January 10 to January 31, 2023. From 2 ancillary analyses of the ICON7 trial with individual patient data and tumor gene expression profiles, an ICON7-A cohort was generated comprising 745 cases. From other studies, published Kaplan-Meier curves were graphically analyzed.

**Exposures:**

Bevacizumab treatment vs placebo or no treatment.

**Main Outcomes and Measures:**

Restricted mean survival time and relative risk of progression at a given time point between bevacizumab treatment and control groups.

**Results:**

In the ICON7-A cohort (n = 745), restricted mean survival analysis showed that bevacizumab treatment (n = 384) had significantly better progression-free survival (PFS) than the control (n = 361) before bevacizumab discontinuation (restricted mean survival time ratio, 1.08; 95% CI, 1.05-1.11; *P* < .001), but had significantly worse PFS after bevacizumab discontinuation (0.79; 95% CI, 0.69-0.90; *P* < .001), showing rebound. In a post hoc analysis, the rebound was similarly observed both in homologous recombination deficiency (HRD) (before, 1.05; 95% CI, 1.02-1.09; *P* < .001; after, 0.79; 95% CI, 0.63-0.98; *P* = .04) and non-HRD tumors (before, 1.08; 95% CI, 1.03-1.15; *P* < .001; after, 0.71; 95% CI, 0.56-0.90; *P* < .001) of the serous subtype, but not in the nonserous subtype (before, 1.11; 95% CI, 1.05-1.18; *P* < .001; after, 0.94; 95% CI, 0.78-1.15; *P* = .57). In Kaplan-Meier curve image–based analysis, the trend of rebound effect was consistently observed in the overall ICON7 and GOG-0218 cohorts and their subgroups stratified by prognostic factors, homologous recombination–associated mutations, and chemotherapy sensitivity. In contrast, no such trend was observed in the studies GOG-0213, OCEANS, AURERIA, and MITO16B, in which patients who experienced relapse received bevacizumab until progression.

**Conclusions and Relevance:**

In ovarian cancer, bevacizumab may reduce progression for approximately 1 year after initiation, but discontinuation may increase subsequent progression in the serous subtype regardless of HRD status. The results suggest that in the first-line treatment, bevacizumab may be more beneficial in patients with a shorter prognosis who are less likely to experience the rebound outcome.

## Key Points

**Question:**

How should bevacizumab be used in the treatment of ovarian cancer?

**Findings:**

This cohort study analyzed published data of randomized phase 3 trials of bevacizumab in ovarian cancer found that the treatment outcomes of bevacizumab changed over time, with a markedly increased risk of tumor progression in the bevacizumab group after a predetermined discontinuation (ie, rebound). No such outcome was noted in settings of recurrent cancer when bevacizumab was continued until progression.

**Meaning:**

The findings of this study suggest that administration of bevacizumab should be considered for its time-dependent effect.

## Teaser

This cohort study examines data from randomized clinical trials on mean survival time and relative risk of progression of ovarian cancer with use of bevacizumab.

## Introduction

Ovarian cancer has the worst prognosis among gynecologic cancers.^1^ Most cases are diagnosed at an advanced stage with peritoneal dissemination and require a combination of surgery and drug therapy. The standard first-line chemotherapy is a combination of paclitaxel and carboplatin, but in the past decade, bevacizumab, an antivascular endothelial growth factor (VEGF)-A antibody, has been incorporated with chemotherapy and used for subsequent maintenance therapy.^2^ However, high-grade serous carcinomas, which make up most of the ovarian cancers, are frequently associated with DNA homologous recombination deficiency (HRD), and since HRD is related to sensitivity to platinum and poly (adenosine diphosphate-ribose) polymerase (PARP) inhibitors,^3,4,5,6^ treatment individualization based on HRD status has recently been proposed in clinical practice.^7^ Therefore, it has become more important to investigate in detail the association between therapeutic effect of bevacizumab and HRD status in ovarian cancer.

In the ICON7 trial, a randomized phase 3 clinical trial of 1528 women with newly diagnosed ovarian cancer who received standard chemotherapy or bevacizumab in combination, bevacizumab was administered at 7.5 mg/m^2^ for a total of 18 cycles (12 months) and reduced the risk of progression with a hazard ratio of 0.81 (95% CI, 0.70-0.94).^8^ In the GOG-0218 study, bevacizumab was administered at 15 mg/m^2^ for a total of 22 cycles (15 months), reducing the risk of progression with a hazard ratio of 0.72 (95% CI, 0.63-0.82).^9^ However, since the difference in progression risk between the comparison arms significantly varied over time, the proportional hazards assumption was known to be inappropriate.^8^ Nevertheless, previous reports examining the use of bevacizumab, including systematic reviews, have still applied the Cox proportional hazards regression model.^10,11^ It should be evaluated by methods that can be adapted when the assumption is not valid, such as restricted mean survival time (RMST) analysis.^12^

Kaplan-Meier (KM) survival curves shown in published studies can be considered as easily accessible, highly informative, and useful research materials. Some of us^13^ previously analyzed KM curves from the ICON7,^8^ GOG-0218,^9^ and BOOST^14^ trials and reported the changes in relative risk of progression between the treatment arms at every 15 months. In recent years, several methods for more detailed image-based analysis of published KM curves and reconstruction of the original data have been reported.^15,16^

Herein we analyze clinical and gene expression data from 2 ancillary analyses of the ICON7^17,18^ trial (ICON7-A cohort) and images of KM curves from all the published phase 3 clinical trials of bevacizumab in both first-line and recurrent ovarian cancer. We then analyze the risk of progression with bevacizumab treatment. The results of the study may provide important insights for optimizing the use of bevacizumab in the treatment of ovarian cancer.

## Methods

This study was exempt from institutional review board approval and patient informed consent because only previously published anonymized data were used. This study adhered to the Strengthening the Reporting of Observational Studies in Epidemiology (STROBE) reporting guideline.

### ICON7-A Cohort

We integrated data sets from 2 independent ancillary analyses of ICON7 by Kommoss et al^17^ and Desbois et al.^18^ We obtained microarray gene expression profiles (DASL, Illumina) and clinical information for 380 cases deposited by Kommoss et al^17^ from the National Center for Biotechnology Information Gene Expression Omnibus (GEO140082). According to the Kommoss et al^17^ study, of a total of 533 patients enrolled in ICON7 from Germany (AGO-OVAR11 trial), 423 had available formalin-fixed paraffin-embedded tumor tissues and 391 had sufficient RNA available for the microarray analysis. Desbois et al^18^ performed total RNA sequencing on 370 formalin-fixed paraffin-embedded tumor tissues collected from the ICON7 trial. Their raw sequencing data with clinical information were obtained through the European Genome-Phenome Archive (accession number EGAS00001003487) and 365 cases for which clinical and sequencing data were matched were included in this study. All 745 patients for whom the above data were available were included in the analysis and their baseline characteristics are summarized in eTable 1 in Supplement 1. Since age data were categorized in the Desbois et al^18^ report, we adopted the classification, with patients aged 65 years or older assigned to the high age category. There were no missing data. Only stratified ages are available and the average age is unknown. Racial information is also unknown.

### Calculation of Progression Risk Curves

The coordinates of the x- and y-axes and each KM curve were extracted from the figures of the articles using ImageJ software, version 1.53t.^19^ Missing parts of the curves were manually completed. The survival rate at each day point on the curve was calculated based on the x-axis coordinates at times 0 and 12 months or 24 months and the y-axis coordinates at survival rates of 0 and 100%. The progression risk at a given time point was calculated as the decrease on the survival curve at 30 days after that time point. The ratio of the progression risk of the treatment group to that of the control group at each time point was calculated as the relative risk. When either was 0, the relative risk was considered to be incalculable. The progression risks and relative risk at each time point were smoothed by simple moving averages over the 60 days before and after the time point, and the changes were analyzed. The sources of the previously published KM curves used in this study are summarized in eTable 2 in Supplement 1.

### Statistical Analysis

Restricted mean survival time of bevacizumab treatment and control groups were compared using the survRM2 package in R, version 4.1.2 (R Foundation for Statistical Computing). Adjusted RMST by integrating an adjusted Kaplan-Meier estimator with inverse probability weighting was performed according to a previous report.^20^ All other statistical analyses and result visualization were performed using Python, version 3.8.8 (Python Software Foundation). Survival analyses including Kaplan-Meier curve, Cox proportional hazards assumption test, and log-rank test were performed using Lifelines, version 0.26.3 in Python. The Spearman rank correlation test was performed using SciPy, version 1.7.2, in Python. Machine learning analyses were performed using Scikit-learn, version 1.0.1, in Python. With 2-sided unpaired testing, the significance threshold was set at *P* < .05.

## Results

### Progression Risk Over Time in ICON7-A Cohort

**Figure 1.**
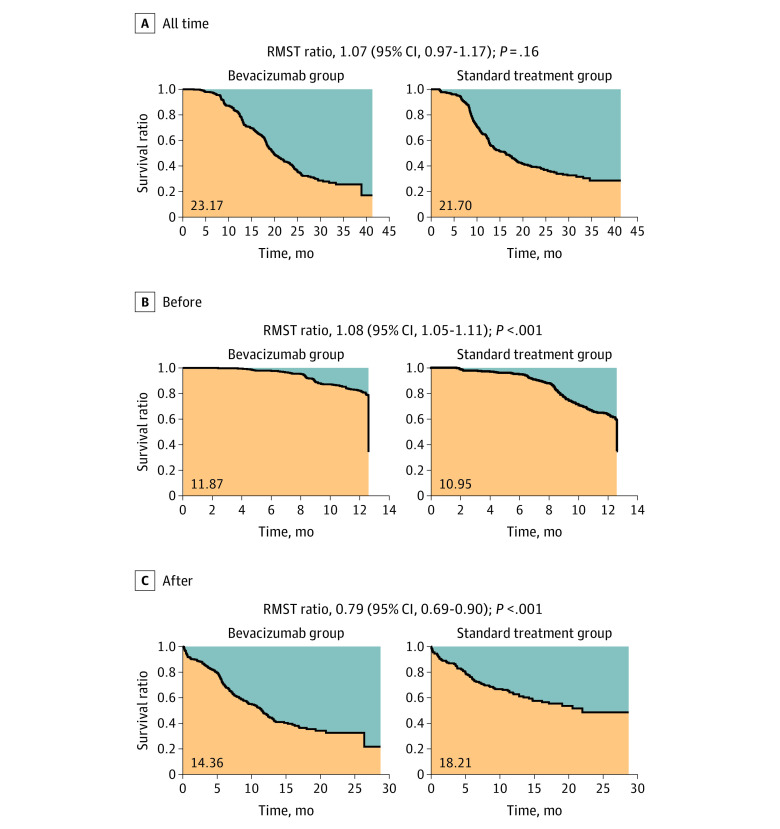
Restricted Mean Survival Time (RMST) Analysis in the ICON7-A cohort The RMST of progression-free survival was compared between the bevacizumab treatment and control groups for the overall period, before bevacizumab discontinuation, and after bevacizumab discontinuation. The differences with RMST were not significant between the bevacizumab treatment and control groups in the overall period, but survival was significantly longer in the bevacizumab group before bevacizumab discontinuation and significantly shorter in the control group after discontinuation.

In the ICON7-A cohort (n = 745) (eTable 1 in Supplement 1), comparative analysis of progression-free survival (PFS) between the bevacizumab group (n = 384) and standard treatment group (control) (n = 361) revealed that the proportional hazards model was not valid (eFigure 1A, B in Supplement 1). We calculated the risk of progression at a given point in time based on the number of patients whose cancer progressed during the following 30 days and the ratio of the risk of progression between the 2 groups (bevacizumab treatment vs control) as the relative risk (eFigure 1C, D in the Supplement). The changes in these values were examined by smoothing with a simple moving averages from before and after the 60 days (eFigure 1E in Supplement 1). The risk of progression in the bevacizumab group was lower than in the control group in the early treatment period, but gradually increased from around 6 months, reached the same level as the control group at around 12 months, when treatment was discontinued, and exceeded the control group thereafter (eFigure 1E in Supplement 1). Restricted mean survival time analysis showed that PFS was not significantly different between the 2 groups in the overall period (RMST ratio, 1.07; 95% CI, 0.97-1.17; *P* = .16), but was significantly better in the bevacizumab  group before bevacizumab discontinuation (RMST ratio, 1.08; 95% CI, 1.05-1.11; *P* < .001), and was significantly worse in the bevacizumab  group after the discontinuation (RMST ratio,  0.79; 95% CI, 0.69-0.90; *P* < .001) (Figure 1). In addition, adjusted RMST analysis^20^ using stage, surgical completion, age, and histologic characteristics as covariates showed similar results (eFigure 2 in Supplement 1). Hereafter, the sharp increase in risk of progression observed after bevacizumab discontinuation is referred to as rebound.

### Analysis of ICON7-A Divided Into Serous and Nonserous Tumors

**Figure 2.**
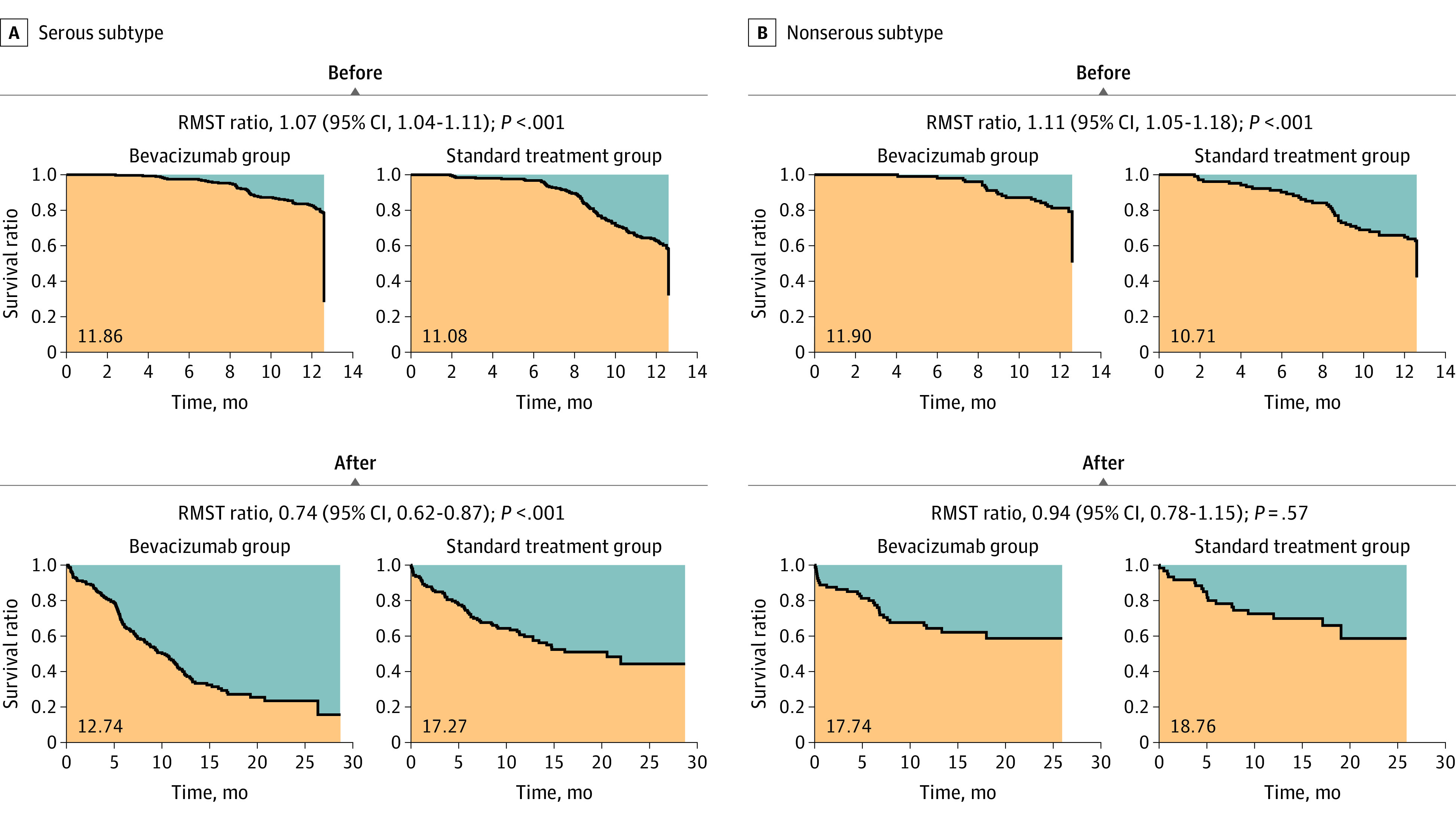
Restricted Mean Survival Time (RMST) Analysis in the ICON7-A Cohort Stratified by Serous and Nonserous Histologic Characteristics A, Analysis in the serous subtype. The reversal of the difference in RMST was observed between before and after bevacizumab discontinuation, similar to that in the overall cohort (Figure 1). B, Analysis in the nonserous subtype. The reversal of the difference in RMST was not significantly observed between before and after bevacizumab discontinuation.

As shown in eFigure 2 in Supplement 1, the serous subtype showed a better PFS before bevacizumab discontinuation but a worse PFS after the discontinuation than the nonserous subtype. We stratified the patients by serous (n = 535) and nonserous (n = 210) subtypes and compared the bevacizumab treatment and control groups. In the serous subtype, the same rebound effect was observed as in the whole cohort (eFigure 3A in Supplement 1); the difference of RSMT and the risk of progression between the bevacizumab and control groups were reversed before and after bevacizumab discontinuation (before: RMST ratio, 1.07; 95% CI, 1.04-1.11; *P* < .001; after: RMST ratio, 0.74; 95% CI, 0.62-0.87; *P* < .001) (Figure 2A). In contrast, in the nonserous subtype, although there was a gradual decrease in the risk of progression during the bevacizumab treatment period in the bevacizumab group (eFigure 3B in Supplement 1), no obvious rebound was observed after the discontinuation (before: RMST ratio, 1.11; 95% CI, 1.05-1.18; *P* < .001; after: RMST ratio, 0.94; 95% CI, 0.78-1.15; *P* = .57) (Figure 2B).

### Analysis of ICON7-A Serous Tumors Divided Into HRD and Non-HRD

**Figure 3.**
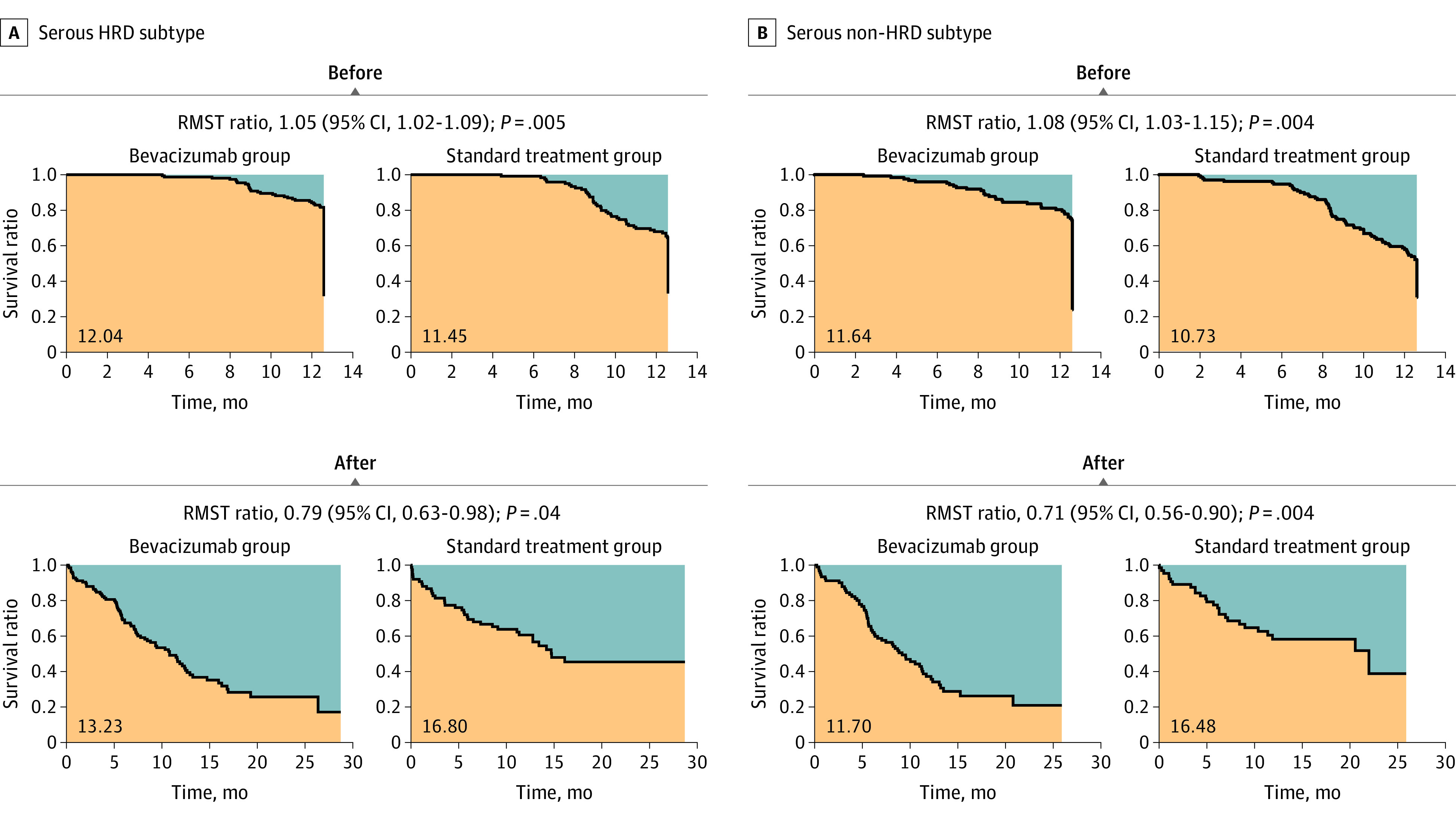
Restricted Mean Survival Time (RMST) Analysis in the ICON7-A Serous Cohort Stratified by Homologous Recombination Deficiency (HRD) and Non-HRD Subtypes A, Analysis in the serous HRD subtype. The reversal of the difference in RMST was observed before and after bevacizumab discontinuation, similar to that in the serous cohort (Figure 2A). B, Analysis in the serous non-HRD subtype. The reversal of the difference in RMST was observed between before and after bevacizumab discontinuation, similar to that in the serous cohort (Figure 2A).

To examine whether the HRD status is associated with bevacizumab treatment in the serous type, we predicted HRD status of patients in the ICON7-A cohort based on their gene expression profiles (eMethods in Supplement 1). We found that approximately half of the patients (279 of 534 [52%]) were assigned to the HRD group and PFS was better in the HRD group than in the non-HRD group (*P* = .04) (eFigure 4A in Supplement 1). Stratified analysis showed that the change in risk of progression over time and the rebound effect associated with bevacizumab treatment were similarly observed both in HRD (before: RMST ratio, 1.05; 95% CI, 1.02-1.09; *P* < .001; after: RMST ratio, 0.79; 95% CI, 0.63-0.98; *P* = .04) (Figure 3A; eFigure 4B in Supplement 1) and in non-HRD cases (before: RMST ratio, 1.08; 95% CI, 1.03-1.15; *P* < .001; after: RMST ratio, 0.71; 95% CI, 0.56-0.90; *P* = .004) (Figure 3B; eFigure 4C in Supplement 1), indicating that HRD status did not appear to be associated with bevacizumab administration.

### Analysis of KM Curves of First-Line Treatment Cases

Next, we developed a method to estimate event risk at each time point by analyzing the images of published KM survival curves (eFigure 5A, B in Supplement 1). The method was applied to the KM curves constructed from individual patient data of the ICON7-A cohort and confirmed to produce a very similar result to the one described above (eFigure 5C, D, eFigure 1D, E in Supplement 1).

**Figure 4.**
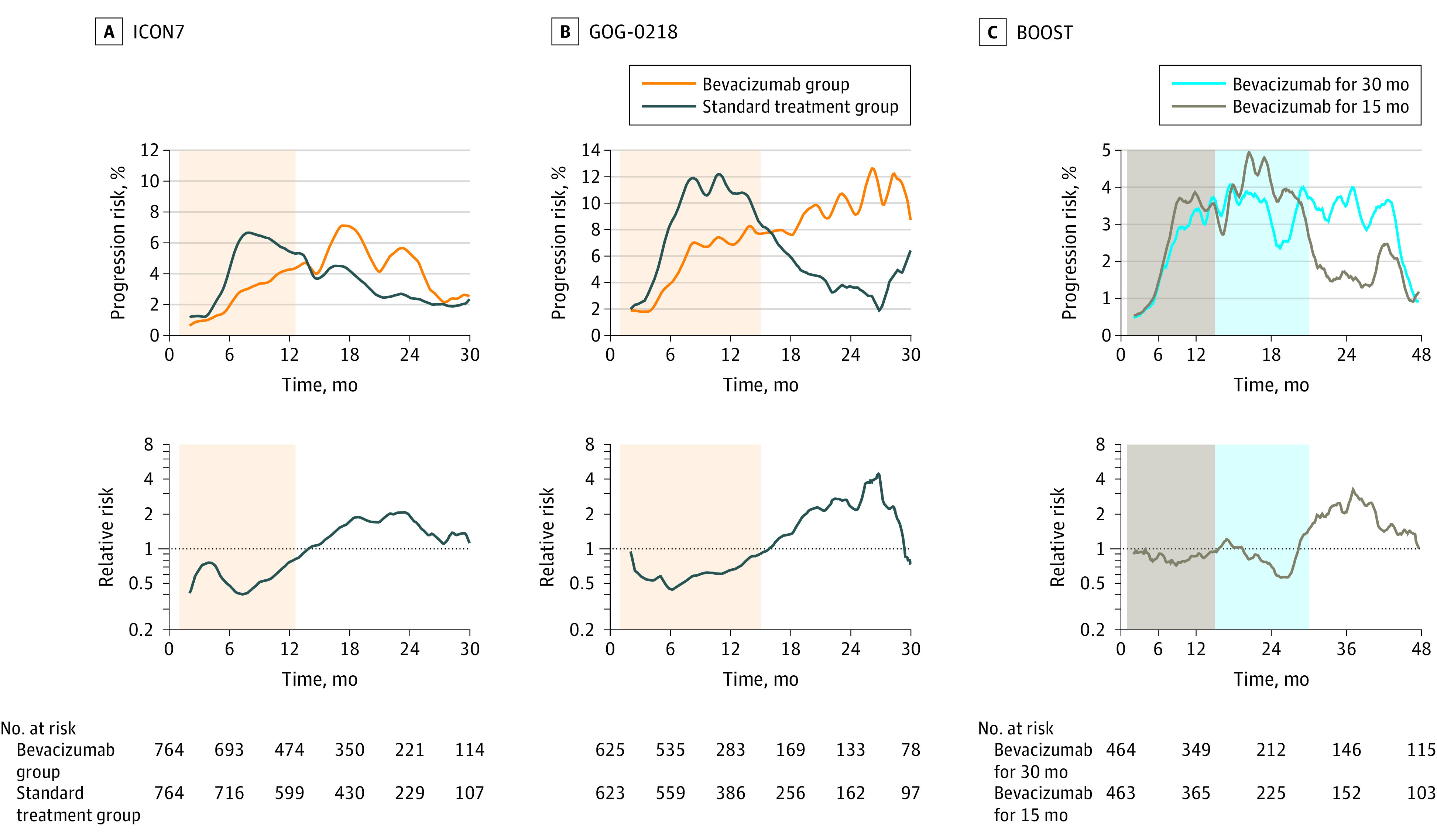
Image-Based Analyses of Kaplan-Meier Curves for Phase 3 Trials With Bevacizumab in the First-Line Setting A, Comparison of the bevacizumab treatment and the control groups in the ICON7 overall cohort. Changes over time in the risk of progression and the relative risk are shown. Time-dependent change in the risk of progression and the rebound effect after discontinuation of bevacizumab were the same as those in the ICON7-A cohort (eFigure 1D in Supplement 1). The shaded area represents the period of bevacizumab administration. B, Analysis in the GOG-0218 overall cohort. The results were similar to those in the ICON7 cohort. C, Comparison of the groups treated with bevacizumab for 30 months and for 15 months in the BOOST trial. The risk of progression in the bevacizumab 30-month group was slightly lower than in the bevacizumab 15-month group from month 15 to month 30, but became higher after month 30. The gray background represents 15 months and blue represents 15 to 30 months of bevacizumab administration.

Using this method, we analyzed the images of the KM curves from the previous phase 3 trials for bevacizumab (eTable 2 in Supplement 1). The results from the original ICON7 cohort^8^ were the same as those from the ICON7-A (Figure 4A; eFigure 1E in Supplement 1). The change was similarly observed both in the high-risk patients, defined as those with International Federation of Gynecology and Obstetrics stage IV disease or with International Federation of Gynecology and Obstetrics stage III disease and more than 1.0 cm of residual disease after debulking surgery, and in the non–high-risk patients (eFigure 6A in Supplement 1). The results from the GOG-0218 trial^9^ were similar to those of the ICON7 trial (Figure 4B). In a subgroup analysis of the GOG-0218 trial,^21^ in which cases were divided by mutation status in homologous recombination repair-related genes, there was no obvious difference in the changes (eFigure 6B in Supplement 1). The results were also similar in another subgroup analysis of GOG-0218 stratified by chemosensitivity status as determined by changes in blood CA125 levels^22^ (eFigure 6C in Supplement 1). In the BOOST trial,^14^ which randomly assigned patients to receive bevacizumab for either 15 or 30 months, the risk of progression in the 30-month group was slightly lower than in the 15-month group from month 15 to month 30, but was higher after month 30 (eFigure 4C in Supplement 1).

### Analysis of KM Curves of Recurrent Cases

**Figure 5.**
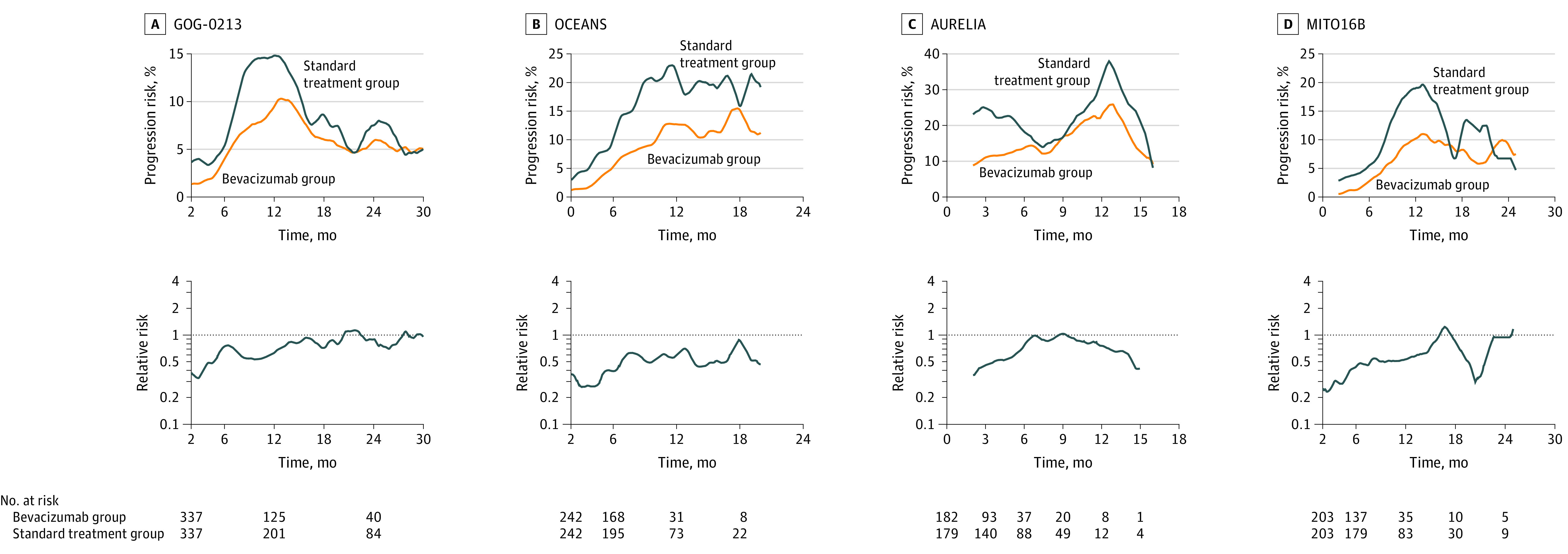
Image-Based Analyses of Kaplan-Meier Curves for Phase 3 Trials With Bevacizumab in Recurrent Setting Changes over time in the risk of progression and the relative risk of the bevacizumab treatment and control groups for the GOG-0213 (A), OCEANS (B), AURELIA (C), and MITO16B (D) trials. In the recurrent cases, bevacizumab treatment was continued until progression, and no rebound effect was observed.

We analyzed KM curves from the phase 3 trials in patients with recurrent ovarian cancer (eTable 2 in Supplement 1). In GOG-0213, bevacizumab was administered in combination with paclitaxel plus carboplatin followed by maintenance therapy for platinum-sensitive recurrence.^23^ In OCEANS, bevacizumab was administered in combination with gemcitabine plus carboplatin followed by maintenance therapy for platinum-sensitive recurrence.^24^ In AURELIA, bevacizumab was used in combination with a nonplatinum monotherapy for platinum-resistant recurrence.^25^ In MITO16B, bevacizumab was administered in combination with platinum-doublet for platinum-sensitive recurrence previously treated with bevacizumab in the first-line setting.^26^ In all of these studies, the duration of bevacizumab administration was not predetermined and was continued until disease progression or an unacceptable adverse event occurred. In common with all these studies, the relative progression risk of the bevacizumab group compared with the control group was lowest soon after the start of treatment and then gradually increased over time, but did not consistently exceed 1, indicating no rebound (Figure 5).

## Discussion

The ICON7-A cohort (n = 745) we compiled in this study had about half the number of cases of the original ICON7 cohort (n = 1528),^8^ and the KM curves for PFS were almost identical to those of the original article (eFigure 1A in Supplement 1). The association of bevacizumab with reduced risk of progression peaked at about 6 months and then disappeared at about 12 months. Even for cancer types other than ovarian cancer, the difference in PFS with bevacizumab is often greatest around 12 months.^2^ The main action of bevacizumab, an anti-VEGF-A antibody, is inhibition of angiogenesis in tumor tissue. Theoretically, it can make tumor tissue hypoxic and hyponutrient and induce apoptosis and necrosis of tumor cells, but it has little or no direct cell-killing effect.^27^ Instead, the hypoxia-induced, VEGF-independent, delayed angiogenesis that would occur during bevacizumab treatment may be responsible for tumor progression and recurrence.^27^ Early studies reported that restoration of vessel structure and function by anti-VEGF antibodies may improve blood perfusion and drug delivery of cytotoxic agents to tumors,^28,29^ but a more recent study reported that the combination of anti-VEGF antibodies rather reduced the intratumor concentrations of cytotoxic agents.^30^ In addition, the combination with bevacizumab in the GOG-0218 study did not show any improvement in response rate.^31^ One study reporting that VEGF had tumor immunosuppressive effects^32^ led to the expectation that anti-VEGF antibodies would activate antitumor immunity, but another study reported that hypoxia induced by anti-VEGF antibodies rather suppressed antitumor immunity.^33^ Collectively, current evidence suggests that the primary action of bevacizumab is presumed to be solely cytostatic, rather than cytotoxic.

In this study, the ICON7-A cohort analysis stratified by serous and nonserous subtypes showed that rebound was only observed in the serous type, but not in the nonserous type (Figure 2; eFigure 3 in Supplement 1). To our knowledge, this is the first report to show differences in the outcomes of bevacizumab between histologic subtypes of ovarian cancer. Bevacizumab is thought to be most effective when tumor cell growth is directly dependent on VEGF-signal,^27^ and in some cases of the serous type, cancer cells have been reported to express high levels of VEGF receptors.^34,35^ In other words, differences in dependence on VEGF among histologic subtypes may be associated with the effectiveness of bevacizumab. In addition, the serous type often responds well to initial chemotherapy but more frequently relapses with increased treatment resistance afterword than the nonserous subtype.^36^ This clinical characteristic of the serous type may also be relevant to the differences in results from the other histologic types.

The results of the subgroup analyses of ICON7 and GOG0218 suggest that the change in the progression risk ratio over time and the rebound were independent of whether the patient was at high or low risk, with or without HRD, and sensitive or resistant to chemotherapy. Given that bevacizumab appears to be effective for only about a year and to have a rebound after its discontinuation, it is likely that the benefit of bevacizumab, including improved survival and quality of life, will only be seen in patients with short survival. Our findings may explain the results of previous studies that bevacizumab did not prolong overall survival in the ICON7 and GOG-0218 overall cohorts^37,38^ but prolonged overall survival in chemotherapy-refractory, high-risk patients.^22,39^

The absence of rebound effect in the studies of recurrent ovarian cancer (eFigure 5A-C in Supplement 1) seems to be attributed to the protocol that did not stop bevacizumab until disease progression.^23,24,25^ A similar result was observed in the MITO-16B trial^26^ in which patients received bevacizumab in the first-line treatment and again in recurrent disease (eFigure 5D in Supplement 1). This suggests that bevacizumab is a simple growth inhibitor and does not induce clonal selection in recurrent tumors in the way cytotoxic agents do.^2^ Given that patients who experience relapse have shorter median PFS and overall survival than those receiving first-line treatment, bevacizumab may provide more benefit to patients with recurrent disease.

In the PAOLA-1 trial, in combination with bevacizumab, olaparib significantly prolonged PFS in patients with HRD compared with placebo.^40^ In patients with no residual tumor after primary debulking surgery, the 2-year PFS in the olaparib group was remarkably favorable: 96% in *BRCA*-mutated cases and 80% in HRD cases with wild-type *BRCA*.^41^ A phase 3 trial is currently under way to evaluate the efficacy of adding bevacizumab in the presence of niraparib.^42^ The results of this study may clarify whether PARP inhibitor maintenance therapy can reduce the progression after bevacizumab discontinuation.

### Limitations

A limitation of this study is that individual patient data for the entire cohorts of the ICON7 and GOG-0218 trials were not available. This may lead to potential bias, especially in the subgroup analysis with small numbers of cases, such as the nonserous type. In addition, the image analysis of the KM curve used in this study did not allow for statistical analysis, so no conclusions can be drawn from a scientifically rigorous perspective. The results of this study need to be verified in future studies.

## Conclusions

The findings of this cohort study suggest that the association of bevacizumab administration with the risk of ovarian cancer progression varies over time. Considering that rebound occurs after completion of bevacizumab in the first-line treatment of serous ovarian cancer, bevacizumab may be most useful for patients who are less likely to be affected by the rebound, ie, those with an expected survival of less than 1 year. The use of bevacizumab in combination with PARP inhibitors needs further investigation.

## Notes

Supplement 1. **eMethods.** HRDness Prediction and Validation in External FFPE Ovarian Tumor Sample **eTable 1.** Baseline Characteristics of the ICON7-A Cohort **eTable 2.** The Sources of the Published Kaplan-Meier Curves Used in this Study **eFigure 1.** Analysis of the ICON7-A Cohort **eFigure 2.** Comparison of PFS Using RMST and ARMST in the ICON7-A Cohort **eFigure 3.** Analysis of the ICON7-A Cohort Stratified by Serous and Nonserous Histology **eFigure 4.** Analysis of the ICON7-A Serous Cohort Stratified by HRD and NonHRD Subtype **eFigure 5.** Validation of the Kaplan-Meier Curve Image-Based Analysis in the ICON7-A Cohort **eFigure 6.** Analyses of Kaplan-Meier Curves for Subgroup Analyses in the ICON7 and GOG-0218 Studies
eReferences

Supplement 2.
Data Sharing Statement
