# Supplementary material for: Time-Dependent Changes in Risk of Progression During Use of Bevacizumab for Ovarian Cancer
Source: JAMA Netw Open. 2023 Aug 2;6(8):e2326834. doi: 10.1001/jamanetworkopen.2023.26834 (PMC10398412; doi:10.1001/jamanetworkopen.2023.26834)
Supplement: Supplement 1. — eMethods. HRDness Prediction and Validation in External FFPE Ovarian Tumor Sample eTable 1. Baseline Characteristics of the ICON7-A Cohort eTable 2. The Sources of the Published Kaplan-Meier Curves Used in this Study eFigure 1. Analysis of the ICON7-A Cohort eFigure 2. Comparison of PFS Using RMST and ARMST in the ICON7-A Cohort eFigure 3. Analysis of the ICON7-A Cohort Stratified by Serous and Nonserous Histology eFigure 4. Analysis of the ICON7-A Serous Cohort Stratified by HRD and NonHRD Subtype eFigure 5. Validation of the Kaplan-Meier Curve Image-Based Analysis in the ICON7-A Cohort eFigure 6. Analyses of Kaplan-Meier Curves for Subgroup Analyses in the ICON7 and GOG-0218 Studies eReferences [file jamanetwopen-e2326834-s001.pdf]

Supplementary Online Content

Takamatsu S, Nakai H, Yamaguchi K, Hamanishi J, Mandai M, Matsumura N. Time-dependent changes in risk of progression during use of bevacizumab for ovarian cancer. *JAMA Netw Open*. 2023;6(8):e2326834. doi:10.1001/jamanetworkopen.2023.26834

eMethods. HRDness Prediction and Validation in External FFPE Ovarian Tumor Sample

eTable 1. Baseline Characteristics of the ICON7-A Cohort

eTable 2. The Sources of the Published Kaplan-Meier Curves Used in this Study

eFigure 1. Analysis of the ICON7-A Cohort

eFigure 2. Comparison of PFS Using RMST and ARMST in the ICON7-A Cohort

eFigure 3. Analysis of the ICON7-A Cohort Stratified by Serous and Nonserous Histology

eFigure 4. Analysis of the ICON7-A Serous Cohort Stratified by HRD and NonHRD Subtype

eFigure 5. Validation of the Kaplan-Meier Curve Image-Based Analysis in the ICON7-A Cohort

eFigure 6. Analyses of Kaplan-Meier Curves for Subgroup Analyses in the ICON7 and GOG-0218 Studies

eReferences

This supplementary material has been provided by the authors to give readers additional information about their work.

© 2023 Takamatsu S et al. *JAMA Network Open.*

eMethods. HRDness prediction and validation in external FFPE ovarian tumor samples

We previously identified a gene expression signature associated with HRD (HRDness signature) in ovarian cancer using the dataset from The Cancer Genome Atlas (TCGA-OV), developed a scoring method to calculate its enrichment for each sample and a machine learning method to predict whether each sample is HRD or non-HRD, using an external gene expression dataset as input [1]. Briefly, TCGA-OV samples were labeled as either HRD or non-HRD based on their genomic scar scores, and the gene expression values of the HRDness signature were used as feature values to build four classifiers using different machine learning methods, namely, k-nearest neighbor, support vector machine, random forest, and linear regression. Input gene expression values for HRDness signature were calibrated against the TCGA-OV dataset using SVA::Combat [2]. In this study, each tumor was assigned to HRD when two or more of the four classifiers predicted HRD; otherwise to non-HRD.

From GEO140082 by Kommes et al [3], normalized gene expression intensity data (GSE140082_geo.normdata.csv.gz) were obtained and used as input data. From EGAS00001003487 by Desbois et al [4], the downloaded paired-end fastq files were preprocessed using fastp [5], mapped on to the human reference genome GRCh38 using STAR-RSEM [6, 7], and gene expression values were quantified as fragments per kilobase of exon per million reads mapped (FPKM) and the values were used as input data.

To validate that the HRDness signature [1] can be applicable to gene expression data derived from FFPE specimens, raw sequencing data of tumor RNA sequencing and normal-tumor paired whole exome sequencing from ovarian serous carcinoma specimens performed by Kang et al. [8] were obtained from the NCBI Sequence Read Archive (PRJNA700673). We personally contacted the authors to identify 61 of these that were derived from FFPE tumor tissues.

Somatic mutation call was performed with Mutect2 using hg38 as the reference genome, referring to the Broad Institute's GATK4 pipeline: Somatic short variant discovery (SNVs + Indels). For the *BRCA1/2* genes, truncating mutations that were not marked as 'benign' or 'VUS' in ClinVar [9] or InterVar [10] were retained. Germline *BRCA1/2* variant call with HaplotypeCaller referring to the Broad Institute's GATK4 pipeline: Germline short variant discovery (SNPs + Indels). Truncating mutations that passed the hard-filtration and were annotated as 'pathogenic' in ClinVar [9] or InterVar [10] were extracted. Using the whole exome data as input, Sequenza [11] and scarHRD [12] were used to calculate the HRD score as the sum of telomeric allelic imbalance (TAI), large-scale state transition (LST), and genomic loss of heterozygosity (LOH) scores. According to the method in the original paper [8], the Signature 3 ratio was calculated from the contribution rate of Signature 3 decomposed by MutationalPatterns [13] using all detected somatic mutations as input and COSMIC version 2 mutational signatures 1, 3, and 5 as reference.

© 2023 Takamatsu S et al. *JAMA Network Open.*

The HRDness signature enrichment score was positively correlated with the HRD score and Signature 3 ratio (r=0.29, 0.39, p=0.021, 0.0019, respectively, below).

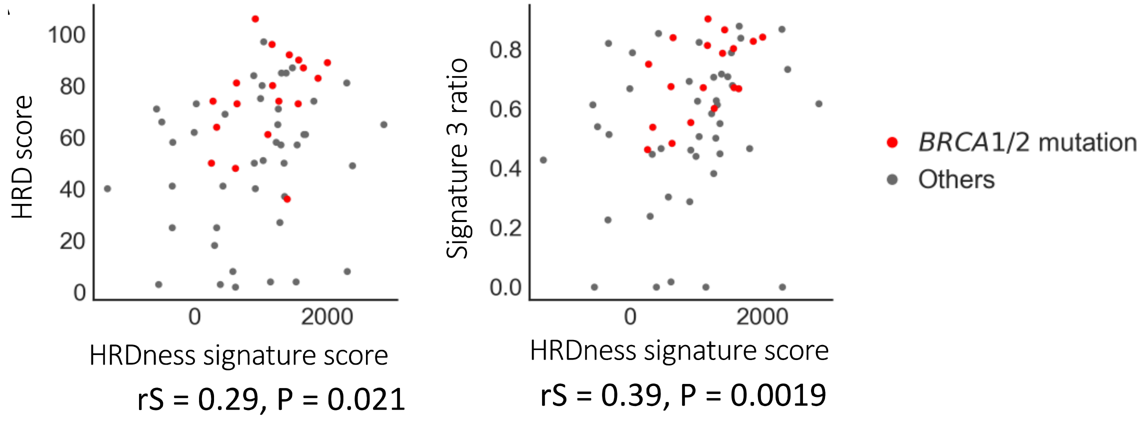

Based on the HRDness prediction, 25 of these 61 cases who received olaparib for platinum-sensitive recurrence were classified into HRD or non-HRD. In these 25 tumors, PFS after olaparib initiation was better in patients with HRD (n=16), including those with *BRCA1/2* mutations and/or those classified as HRD on gene expression, than in those without (n=9) (p=0.0146, below).

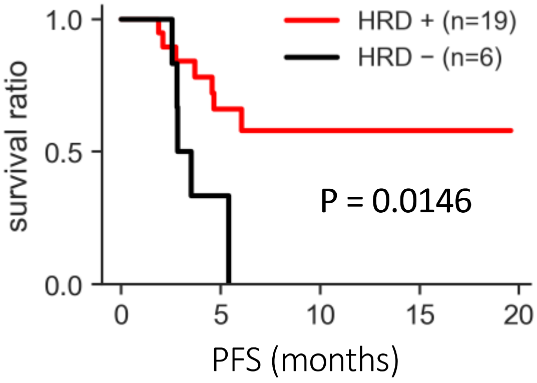

© 2023 Takamatsu S et al. *JAMA Network Open.*

eTable 1. Baseline characteristics of the ICON7-A cohort

	Standard (n=361)	Bevacizumab (n=384)
**Age, n (%)**		
18-39	19 (5)	18 (5)
40-64	244 (68)	261 (68)
>=65	98 (27)	105 (27)
**FIGO Stage, n (%)**		
I	24 (7)	25 (7)
II	34 (9)	43 (11)
III	255 (71)	264 (69)
IV	48 (13)	52 (14)
**Optimal surgery, n (%)**		
Yes	254 (70)	286 (74)
No	104 (30)	97 (26)
No data	3 (1)	1 (0)
**Serous subtype, n (%)**		
Yes	255 (71)	280 (73)
No	106 (29)	104 (27)

© 2023 Takamatsu S et al. *JAMA Network Open.*

eTable 2. The sources of the published Kaplan-Meier curves used in this study.

Figures	Study cohort		Group	Number	Author, journal, year, original figure	PMID
Figure 4A	ICON7	overall	C+T<br>C+T+Bev 12	764<br>764	Perren, NEJM, 2011<br>Figure 3A	22204725
eFigure 5A		high risk	C+T<br>C+T+Bev 12	234<br>231	Perren, NEJM, 2011<br>Figure 4Sb	22204725
		non-high risk	C+T<br>C+T+Bev 12	530<br>533		
Figure 4B	GOG0218	overall	C+T<br>C+T+Bev 15	625<br>623	Burger, NEJM, 2011<br>Figure 2B	22204724
eFigure 5B		with HR mutations	C+T<br>C+T+Bev 15	108<br>120	Norquist, CCR, 2018<br>Figure 1A/1B	29191972
		without HR mutations	C+T<br>C+T+Bev 15	300<br>281		
eFigure 5C		KELIM favorable	C+T<br>C+T+Bev 15	218<br>238	You, JCO, 2022<br>FIG 1	36252167
		KELIM unfavorable	C+T<br>C+T+Bev 15	327<br>304		
Figure 4C	BOOST		C+T+Bev 15<br>C+T+Bev 30	464<br>463	Pfisterer, JCO, 2022<br>Fig 2A	36332161
Figure 5A	GOG0213		C+T<br>C+T+Bev	337<br>337	Coleman, Lancet Oncol, 2017<br>Figure 4	28438473
Figure 5B	OCEANS		C+G+Placebo<br>C+G+Bev	242<br>242	Aghajanian, JCO, 2012<br>Fig 2	22529265
Figure 5C	AURELIA		T/D/TPT<br>T/D/TPT+Bev	182<br>179	Pujade-Lauraine, JCO, 2014<br>Fig 2	24637997
Figure 5D	MITO16b		C+T/G/D<br>C+T/G/D+Bev	203<br>203	Pignata, Lancet Oncol, 2021<br>Figure 2	33539744

Bev: bevacizumab, T: paclitaxel, C: carboplatin, G: gemcitabine, D: pegylated liposomal doxorubicin, TPT: topotecan

© 2023 Takamatsu S et al. *JAMA Network Open.*

eFigure 1. Analysis of the ICON7-A cohort

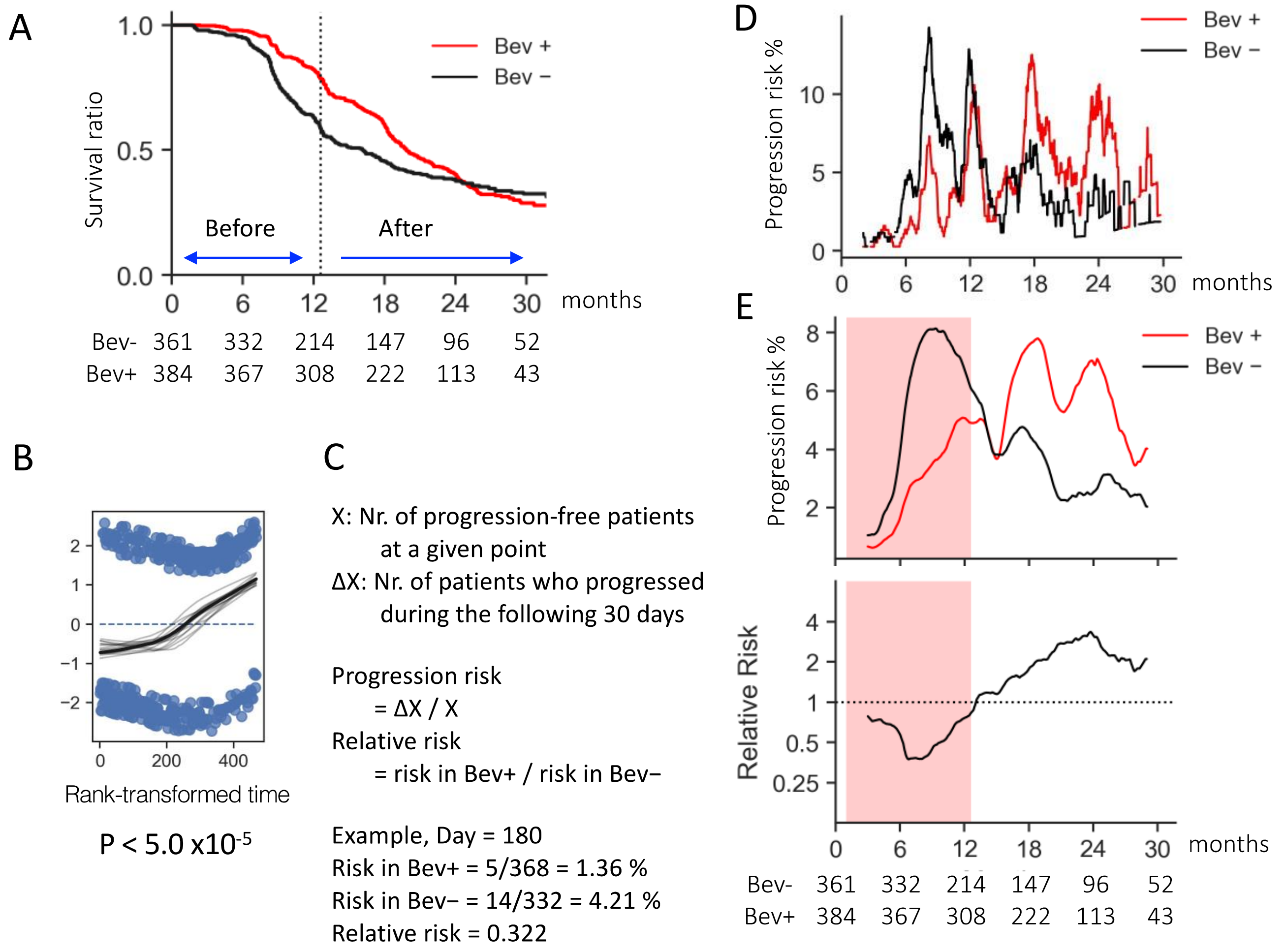

A) Kaplan-Meier survival curves stratified by bevacizumab treatment. The dotted line represents the time of bevacizumab discontinuation. The observation period was divided before and after bevacizumab discontinuation. Bev+: bevacizumab treatment group, Bev-: control group

B) Test of proportional hazard assumption in the Cox model by the Schoenfeld residuals plot. The hazard ratio between the two groups stratified by bevacizumab treatment is not consistent over time.

C) Calculation of the risk of progression at a given time point. For example, based on individual patient clinical data, 368 and 332 patients in Bev+ and Bev- were progression-free at day 180, respectively. From that point to 30 days later, 5 and 14 patients had a relapse. Cases censored without recurrence were excluded in both groups. The risk of progression was calculated to be 5/368 (=1.36%) in Bev+ and 14/328 (=4.27%) in Bev- and the relative risk was (5/368)/(14/332) = 0.322.

D) Plot of the risk of progression per day in Bev+ and Bev- groups.

E) Moving averages of the progression risk (upper) and relative risk (lower) between Bev+ and Bev-. Simple moving averages at 60 days before and after were used at each time point. The red background color represents the period of bevacizumab administration.

© 2023 Takamatsu S et al. *JAMA Network Open.*

eFigure 2. Comparison of PFS using RMST and ARMST in the ICON7-A cohort

		RMST				ARMST			
Period	Factor	Arm1	Arm0	RMST ratio [95%CI]	P-value	Arm1	Arm0	ARMST ratio [95%CI]	P-value
**All time**	Bevacizumab	23.17	21.70	1.07 [0.97,1.17]	.16	23.01	21.98	1.05 [0.96,1.15]	.33
	Stage III/IV	19.45	33.23	0.59 [0.54,0.63]	$<10^{-6}$	19.72	32.87	0.6 [0.52,0.69]	$<10^{-6}$
	Optimal surgery	24.19	15.71	1.54 [1.4,1.7]	$<10^{-6}$	23.39	16.55	1.41 [1.22,1.64]	4.0 x^{-6}
	Age >= 65	19.17	22.79	0.84 [0.76,0.93]	5.6 x^{-4}	20.17	22.41	0.9 [0.81,1.0]	.42
	Serous subtype	21.44	25.27	0.85 [0.77,0.94]	.0011	22.39	22.17	1.01 [0.91,1.13]	.86
**Before**	Bevacizumab	11.87	10.97	1.08 [1.05,1.11]	$<10^{-6}$	11.86	11.01	1.08 [1.05,1.11]	$<10^{-6}$
	Stage III/IV	11.27	12.27	0.92 [0.89,0.94]	$<10^{-6}$	11.28	12.09	0.93 [0.88,0.99]	.33
	Optimal surgery	11.69	10.78	1.08 [1.05,1.12]	8.3 x^{-6}	11.63	10.59	1.1 [1.03,1.17]	.0022
	Age >= 65	11.16	11.55	0.97 [0.93,1.0]	.054	11.24	11.52	0.98 [0.94,1.01]	.17
	Serous subtype	11.49	11.31	1.02 [0.98,1.05]	.36	11.56	11.10	1.04 [1.0,1.08]	.033
**After**	Bevacizumab	14.36	18.21	0.79 [0.69,0.9]	5.9 x^{-4}	14.62	17.78	0.82 [0.72,0.94]	.0052
	Stage III/IV	12.74	22.43	0.57 [0.51,0.63]	$<10^{-6}$	13.24	22.50	0.59 [0.5,0.69]	$<10^{-6}$
	Optimal surgery	16.63	9.20	1.81 [1.47,2.22]	$<10^{-6}$	16.00	11.78	1.36 [0.98,1.88]	.065
	Age >= 65	12.79	15.69	0.82 [0.69,0.96]	.14	13.87	15.38	0.9 [0.77,1.06]	.21
	Serous subtype	14.48	19.79	0.73 [0.64,0.84]	4.8 x^{-6}	15.36	17.11	0.9 [0.77,1.05]	.18

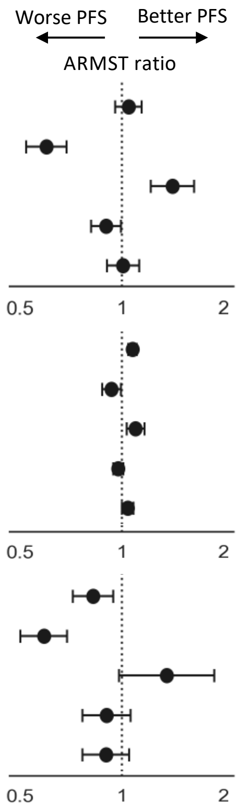

After adjusting covariates, including stage, surgical completion, age, and histology, reversal of the difference in RMST and ARMST before and after bevacizumab discontinuation remained significant.

RMST: restricted mean survival time, ARMST: adjusted restricted mean survival time
Arm1: Estimated RMST or ARMST for factor-positive group
Arm0: Estimated RMST or ARMST for factor-negative group

© 2023 Takamatsu S et al. *JAMA Network Open.*

eFigure 3. Analysis of the ICON7-A cohort stratified by serous and non-serous histology

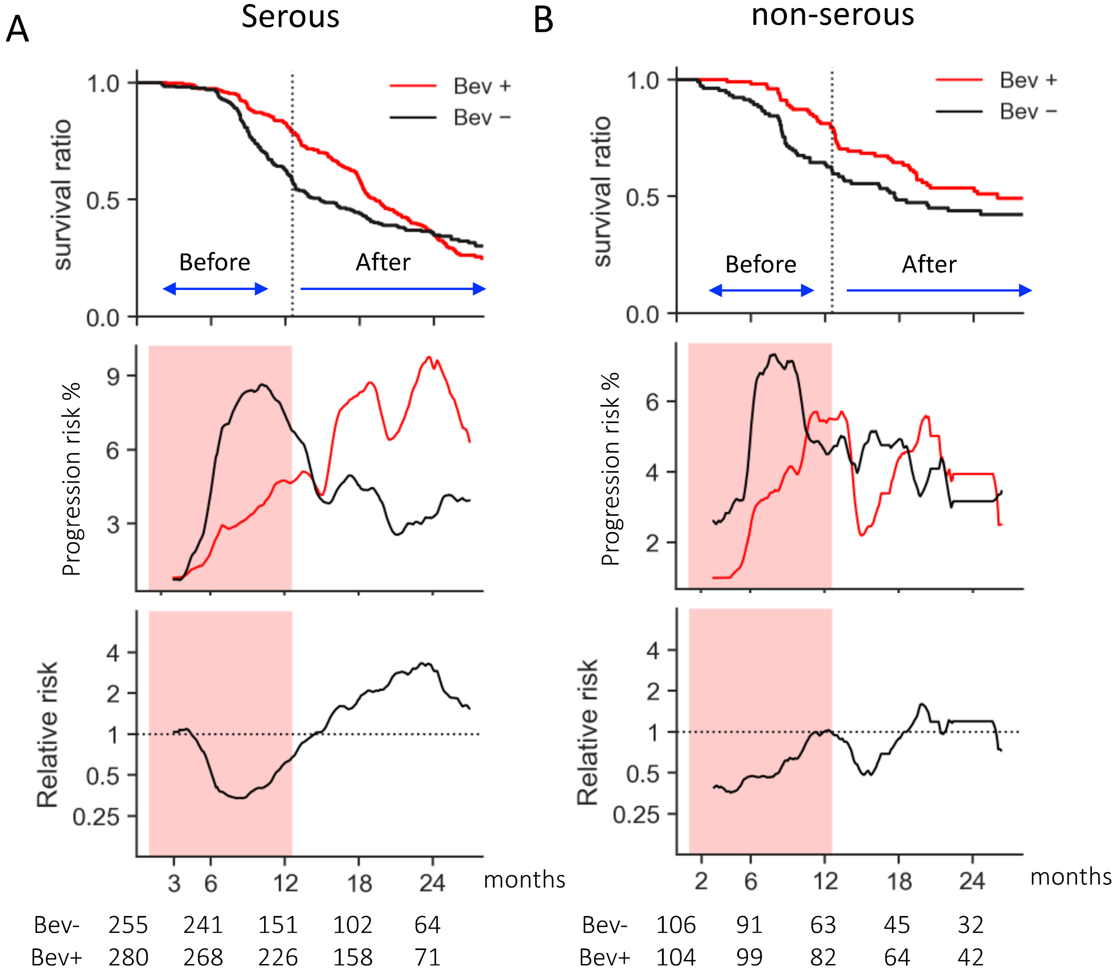

Kaplan-Meier curves for PFS (top), changes over time in the risk of progression (upper) and the relative risk (lower) between bevacizumab treatment (Bev+) vs control (Bev-) groups.

A) Time-dependent changes in the risk of progression and the rebound effect were observed in the serous type, similar to the overall ICON7-A cohort (eFigure1E)

B) On the other hand, there was no obvious rebound effect in the non-serous type. The red background color represents the period of bevacizumab administration.

© 2023 Takamatsu S et al. *JAMA Network Open.*

eFigure 4. Analysis of the ICON7-A serous cohort stratified by HRD and nonHRD subtype

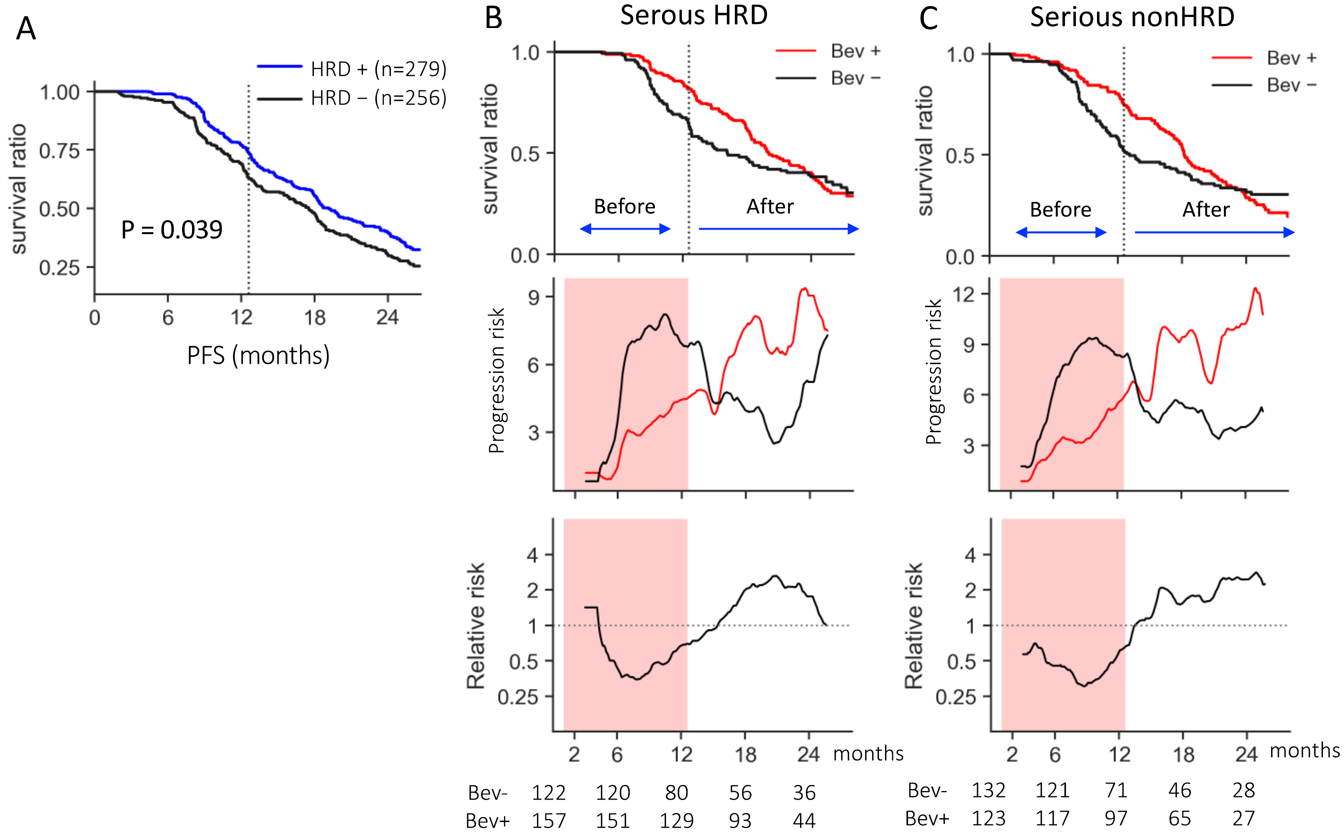

A) Survival analysis for PFS between groups of ICON7-A cohort divided by the HRDness prediction. In the ICON7-A serous cohort, tumors classified as HRD using the HRDness prediction had a better prognosis than the others. P; p-value based on the log-rank test.

B) Kaplan-Meier curves for PFS (top), changes over time in the risk of progression (middle) and the relative risk (bottom) between bevacizumab treatment (Bev+) vs control (Bev-) group in the serous HRD subtype.

C) Kaplan-Meier curves for PFS (top), changes over time in the risk of progression (middle) and the relative risk (bottom) between bevacizumab treatment (Bev+) vs control (Bev-) group in the serous non-HRD subtype.
Time-dependent changes in the risk of progression and the rebound effect were similar to the overall cohort (Figure 2B) in both serous HRD and nonHRD tumors.
The red background color represents the period of bevacizumab administration.

© 2023 Takamatsu S et al. *JAMA Network Open.*

eFigure 5. Validation of the Kaplan-Meier curve image-based analysis in the ICON7-A cohort

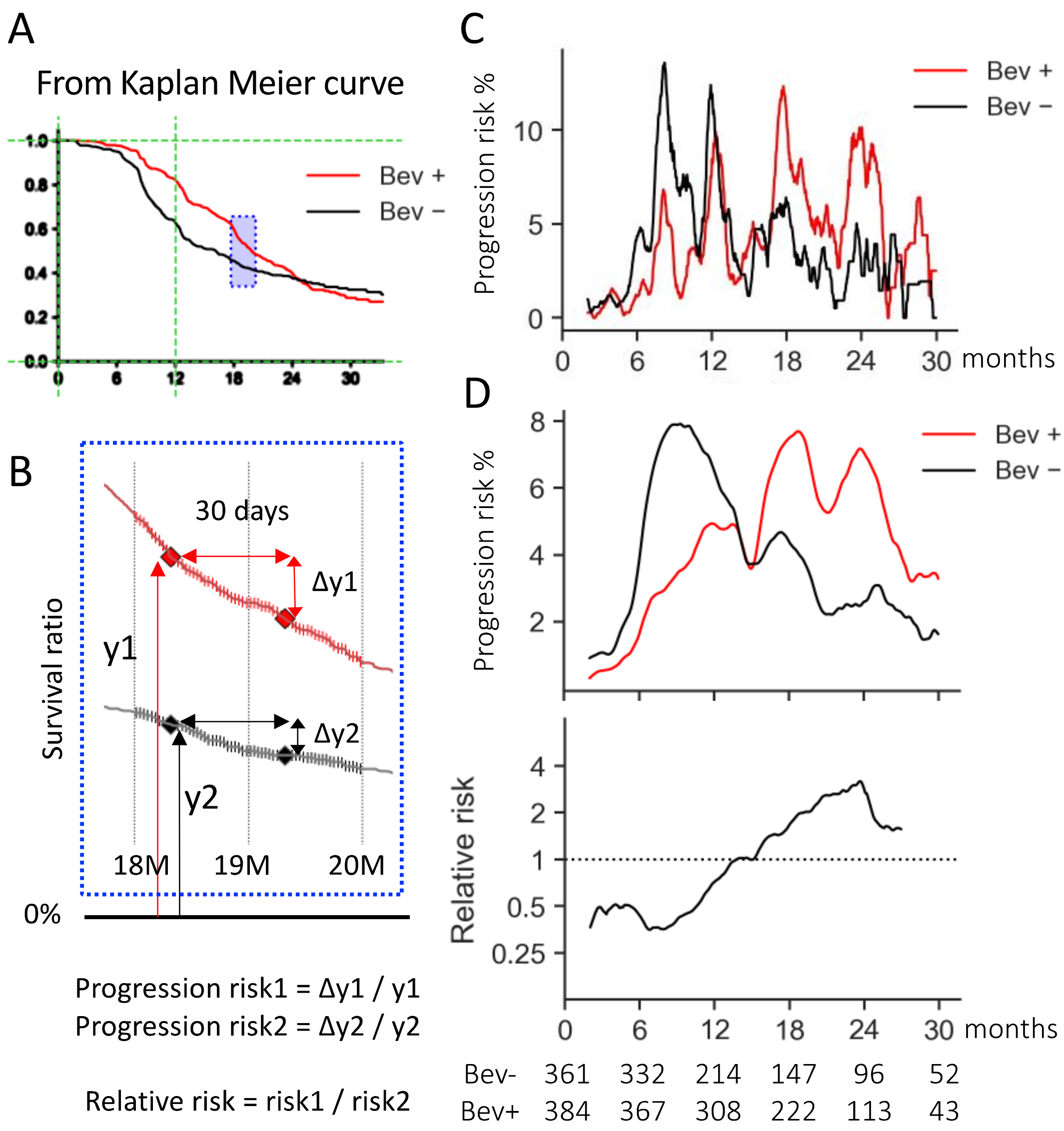

A) Reconstruction of Kaplan Meier curves using Image J.
The survival rates at each day point on the KM curves of the bevacizumab (Bev+) and the control group (Bev-) were calculated based on the X-axis coordinates at time 0 and 12 months and the Y-axis coordinates at survival rates of 0 and 100%.

B) Calculation of the risk of progression at a given point in time: example at one day after 18 months (enlarged view of the area with blue background in A).
The progression risk at a given time point was calculated as the decrease on the survival curve at 30 days after that time point. The relative risk was calculated as the ratio of the progression risk of the subject group to the control group at each time point.

C) Plot of the risk of progression per day in Bev+ and Bev-.

D) Moving averages of the progression risk (upper) and relative risk (lower) between Bev+ and Bev-.
Simple moving averages at 60 days before and after were used at each time point.

© 2023 Takamatsu S et al. *JAMA Network Open.*

eFigure 6. Analyses of Kaplan-Meier curves for subgroup analyses in the ICON7 and GOG-0218 studies

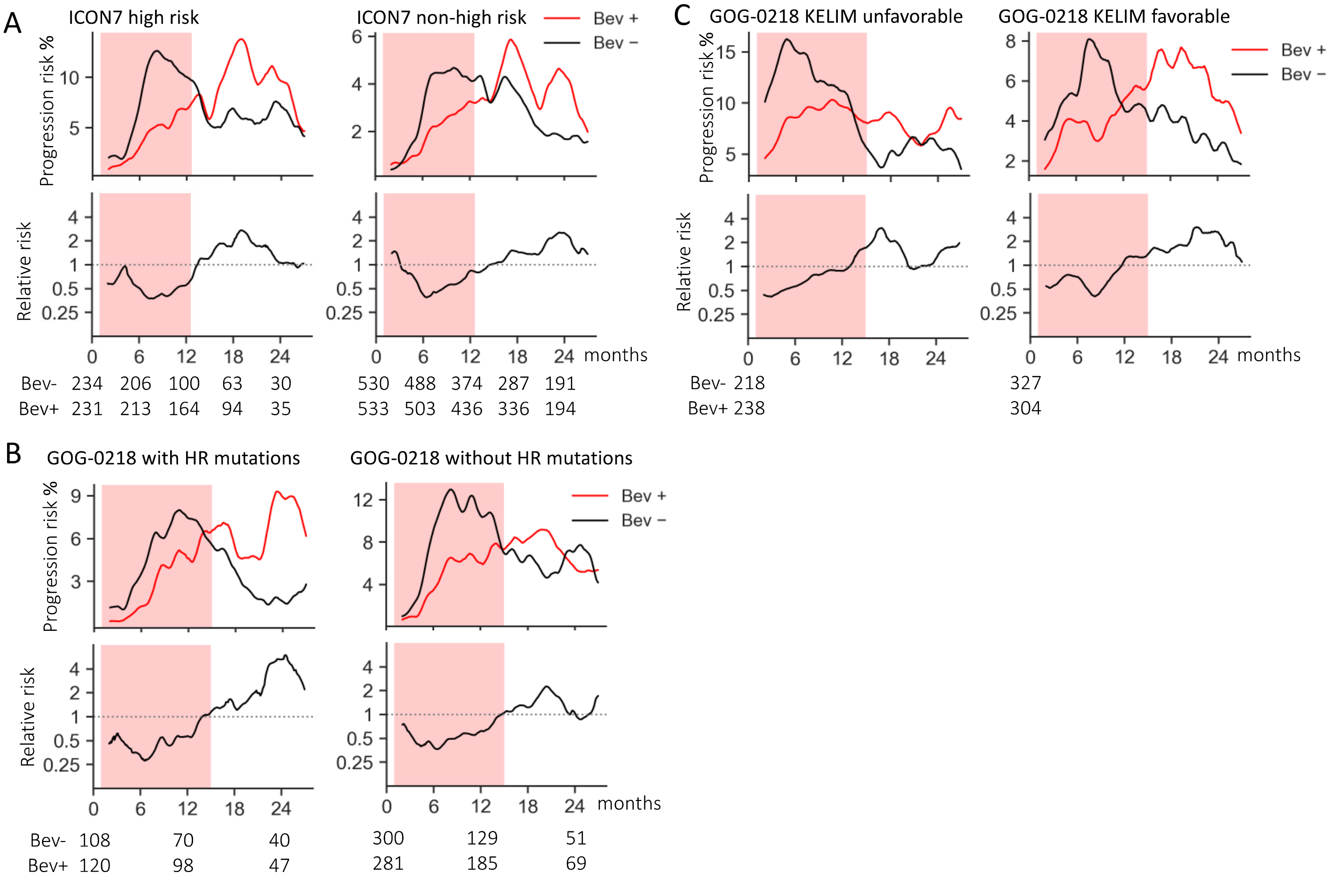

A) Comparison of the bevacizumab (Bev+) and the control (Bev-) groups in the high risk (left) and non-high-risk (right) cases in the ICON7 cohort.
B) GOG-0218 cases with (left) and without (right) homologous recombination repair-related gene mutations.
C) GOG-0218 cases with low (left) and high (right) chemotherapy sensitivity based on serum CA125.

Changes over time in the risk of progression (upper) and the relative risk (lower) are shown. Time-dependent change in the risk of progression and the rebound effect after discontinuation of bevacizumab were similar to those in their original cohorts (Figure 4). The red background color represents the period of bevacizumab administration.

© 2023 Takamatsu S et al. *JAMA Network Open.*

eReferences

1. Takamatsu S, Yoshihara K, Baba T, Shimada M, Yoshida H, Kajiyama H, et al. Prognostic relevance of HRDness gene expression signature in ovarian high-grade serous carcinoma; JGOG3025-TR2 study. British journal of cancer DOI:10.1038/s41416-022-02122-9. PMID:36593360
2. Leek JT, Johnson WE, Parker HS, Jaffe AE, Storey JD. The sva package for removing batch effects and other unwanted variation in high-throughput experiments. Bioinformatics (Oxford, England) 2012;28(6):882-3. DOI:10.1093/bioinformatics/bts034. PMID:22257669
3. Kommoss S, Winterhoff B, Oberg AL, Konecny GE, Wang C, Riska SM, et al. Bevacizumab May Differentially Improve Ovarian Cancer Outcome in Patients with Proliferative and Mesenchymal Molecular Subtypes. Clinical cancer research : an official journal of the American Association for Cancer Research 2017;23(14):3794-801. DOI:10.1158/1078-0432.CCR-16-2196. PMID:28159814
4. Desbois M, Udyavar AR, Ryner L, Kozlowski C, Guan Y, Dürrbaum M, et al. Integrated digital pathology and transcriptome analysis identifies molecular mediators of T-cell exclusion in ovarian cancer. Nature communications 2020;11(1):5583. DOI:10.1038/s41467-020-19408-2. PMID:33149148
5. Chen S, Zhou Y, Chen Y, Gu J. fastp: an ultra-fast all-in-one FASTQ preprocessor. Bioinformatics (Oxford, England) 2018;34(17):i884-i890. DOI:10.1093/bioinformatics/bty560. PMID:30423086
6. Dobin A, Davis CA, Schlesinger F, Drenkow J, Zaleski C, Jha S, et al. STAR: ultrafast universal RNA-seq aligner. Bioinformatics (Oxford, England) 2013;29(1):15-21. DOI:10.1093/bioinformatics/bts635. PMID:23104886
7. Li B, Dewey CN. RSEM: accurate transcript quantification from RNA-Seq data with or without a reference genome. BMC bioinformatics 2011;12:323. DOI:10.1186/1471-2105-12-323. PMID:21816040
8. Kang HG, Hwangbo H, Kim MJ, Kim S, Lee EJ, Park MJ, et al. Aberrant Transcript Usage Is Associated with Homologous Recombination Deficiency and Predicts Therapeutic Response. Cancer research 2022;82(1):142-54. DOI:10.1158/0008-5472.CAN-21-2023. PMID:34711610
9. Landrum MJ, Lee JM, Benson M, Brown GR, Chao C, Chitipiralla S, et al. ClinVar: improving access to variant interpretations and supporting evidence. Nucleic acids research 2018;46(D1):D1062-D1067. DOI:10.1093/nar/gkx1153. PMID:29165669
10. Li Q, Wang K. InterVar: Clinical Interpretation of Genetic Variants by the 2015 ACMG-AMP Guidelines. American journal of human genetics 2017;100(2):267-80. DOI:10.1016/j.ajhg.2017.01.004. PMID:28132688
11. Favero F, Joshi T, Marquard AM, Birkbak NJ, Krzystanek M, Li Q, et al. Sequenza: allele-specific copy number and mutation profiles from tumor sequencing data. Annals of oncology : official journal of the European Society for Medical Oncology 2015;26(1):64-70. DOI:10.1093/annonc/mdu479. PMID:25319062
12. Sztupinszki Z, Diossy M, Krzystanek M, Reiniger L, Csabai I, Favero F, et al. Migrating the SNP array-based homologous recombination deficiency measures to next generation sequencing data of breast cancer. NPJ breast cancer 2018;4:16. DOI:10.1038/s41523-018-0066-6. PMID:29978035
13. Blokzijl F, Janssen R, van Boxtel R, Cuppen E. MutationalPatterns: comprehensive genome-wide analysis of mutational processes. Genome medicine 2018;10(1):33. DOI:10.1186/s13073-018-0539-0. PMID:29695279

© 2023 Takamatsu S et al. *JAMA Network Open.*
