# Supplementary material for: Time-Dependent Changes in Risk of Progression During Use of Bevacizumab for Ovarian Cancer
Source: JAMA Netw Open. 2023 Aug 2;6(8):e2326834. doi: 10.1001/jamanetworkopen.2023.26834 (PMC10398412; doi:10.1001/jamanetworkopen.2023.26834)
Supplement: Supplement 2. — Data Sharing Statement [file jamanetwopen-e2326834-s002.pdf]

Data Sharing Statement

Takamatsu. Time-Dependent Changes in Risk of Progression During Use of Bevacizumab for Ovarian Cancer. *JAMA Netw Open*. Published August 02, 2023.
doi:10.1001/jamanetworkopen.2023.26834

Data
Data available: No

Additional Information
Explanation for why data not available: All data used in this study were processed from publicly available data. The sources of original data compiled from them are included in the Supplement1. The processed data and codes used for the analysis are available on the GitHub project page (https://github.com/shirotak/ov_bev_risk_hrd). Shiro Takamatsu and Noriomi Matsumura had full access to all the data in the study and takes responsibility for the integrity of the data and the accuracy of the data analysis.
